# Development and validation of a risk prediction model and nomogram for colon adenocarcinoma based on methylation-driven genes

**DOI:** 10.18632/aging.203179

**Published:** 2021-06-28

**Authors:** Liangyu Zhu, Hongyu Sun, Guo Tian, Juan Wang, Qian Zhou, Pu Liu, Xuejiao Tang, Xinrui Shi, Lei Yang, Guangjie Liu

**Affiliations:** 1 Department of Epidemiology and Statistics, School of Public Health, Hebei Key Laboratory of Environment and Human Health, Hebei Medical University, Shijiazhuang 050017, P.R. China; 2 Department of Medical Record, The Fourth Hospital of Hebei Medical University, Shijiazhuang 050011, P.R. China; 3 Department of Pathology, The Second Hospital of Hebei Medical University, Shijiazhuang 050000, P.R. China; 4 Department of Clinical Pharmacology, The Fourth Hospital of Hebei Medical University, Shijiazhuang 050011, P.R. China; 5 Department of Thoracic Surgery, The Fourth Hospital of Hebei Medical University, Shijiazhuang 050011, P.R. China

**Keywords:** DNA methylation, colon adenocarcinoma, risk prediction model, nomogram, TCGA

## Abstract

Evidence suggests that abnormal DNA methylation patterns play a crucial role in the etiology and pathogenesis of colon adenocarcinoma (COAD). In this study, we identified a total of 97 methylation-driven genes (MDGs) through a comprehensive analysis of the Cancer Genome Atlas (TCGA) and Gene Expression Omnibus (GEO) databases. Univariate Cox regression analysis identified four MDGs (*CBLN2*, *RBM47*, *SLCO4C*1, and *TMEM220*) associated with overall survival (OS) in COAD patients. A risk prediction model was then developed based on these four MDGs to predict the prognosis of COAD patients. We also created a nomogram that incorporated risk scores, age, and TNM stage to promote a personalized prediction of OS in COAD patients. Compared with the traditional TNM staging system, our new nomogram was better at predicting the OS of COAD patients. In cell experiments, we confirmed that the mRNA expression levels of *CLBN2* and *TMEM220* were regulated by the methylation of their promoter regions. Moreover, immunohistochemistry showed that *CBLN2* and *TMEM220* were potential prognostic biomarkers for COAD patients. In summary, we have established a risk prediction model and nomogram that might be effectively utilized to promote the prediction of OS in COAD patients.

## INTRODUCTION

Colon adenocarcinoma (COAD) is a common global cancer, and has the third highest incidence rate and the second highest mortality rate in the world [1]. A large number of studies have revealed that the occurrence and progression of COAD is associated with a variety of complex factors, such as diet, lifestyle, and genetics [2–4]. Moreover, the rate of early diagnosis of COAD is low, and most patients are diagnosed with advanced disease, so current prognosis of COAD in patients is not satisfactory [5]. Colectomy and neoadjuvant chemoradiotherapy are main treatments for COAD. Unfortunately, the five-year relative survival rate for persons with COAD is only 65% [6]. Therefore, biomarkers with high sensitivity and strong specificity are urgently needed for early diagnosis, survival prediction, and even early treatment of COAD.

As an important part of epigenetics, DNA methylation is an important molecular mechanism associated with human tumorigenesis. In particular, an abnormal methylation pattern in the promoter region of cancer-related genes is related to the diagnosis and prognosis of many types of cancers [7–10]. Additionally, previous studies have shown that methylated mRNA may be a valid predictor of COAD [11, 12]. Chae et al. reported that *FOXO1* hypermethylation could modulate COAD cell proliferation and apoptosis [13]. Zhao et al. revealed that the abnormal methylation of the *CXCL3* and *CXCL8* promoter regions was associated with the poor prognosis of patients with COAD [14]. However, as far as we know, there have been few studies that have integrated clinical data and multiscale omics data to predict the prognosis of COAD, and long-term efforts are still needed [15, 16].

The Cancer Genome Atlas (TCGA) project and the Gene Expression Omnibus (GEO) database have collected a great quantity of cancer-related histochemical data and patients’ clinical data, and provide a large amount of data for researchers to explore the prognosis and biomarkers of various malignant tumors. In this study, we integrated methylation and mRNA expression profiling data from the TCGA and GEO databases, identifying methylation-driven genes (MDGs) related to COAD prognosis, and with these, we established a risk prediction model. In addition, we combined risk score and clinical variables to establish a nomogram to individualize the prediction of the overall survival (OS) of COAD patients. At the same time, we verified that two genes from our risk prediction model (*CBLN2* and *TMEM220*) were silenced by promoter region methylation in colon cells. Finally, through immunohistochemistry, *CBLN2* and *TMEM220* were shown to be potential prognostic biomarkers of COAD.

## RESULTS

### Identification of aberrantly methylated and differentially expressed genes in COAD

**Figure 1:**
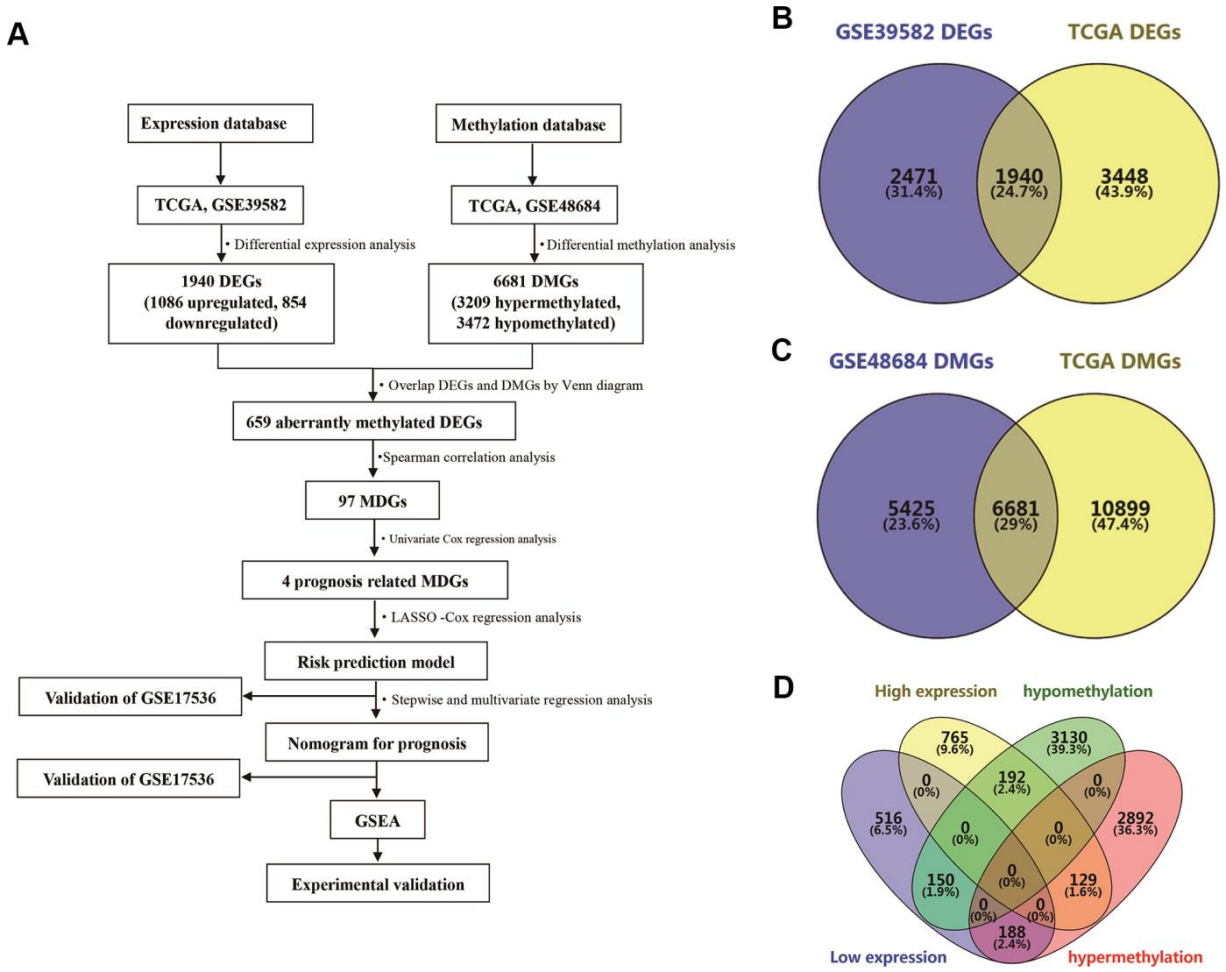
**Identification of differentially expressed genes (DEGs) and differentially methylated genes (DMGs) in colon adenocarcinoma (COAD).** (**A**) Flowchart showing overall design and analytic procedure of this study. (**B**) Overlapping of DEGs from TCGA and GSE39582. (**C**) Overlapping of DMGs from TCGA and GSE48684. (**D**) Identification of aberrantly methylated DEGs.

An analysis flow chart of our bioinformatics workflow is shown in Figure 1A. A total of 1940 differentially expressed genes (DEGs) were detected by overlapping date from the TCGA database and GSE39582 (Figure 1B). Similarly, 6681 differentially methylated genes (DMGs) were identified by overlapping TCGA data and GSE48684 (Figure 1C). Subsequently, we overlapped these DEGs and DMGs, and identified 659 aberrantly methylated DEGs, including 129 genes with high expression and hypermethylation, 188 genes with low expression and hypermethylation, 192 genes with high expression and hypomethylation, and 150 genes with low expression and hypomethylation (Figure 1D).

### Identification of MDGs in COAD

**Figure 2:**
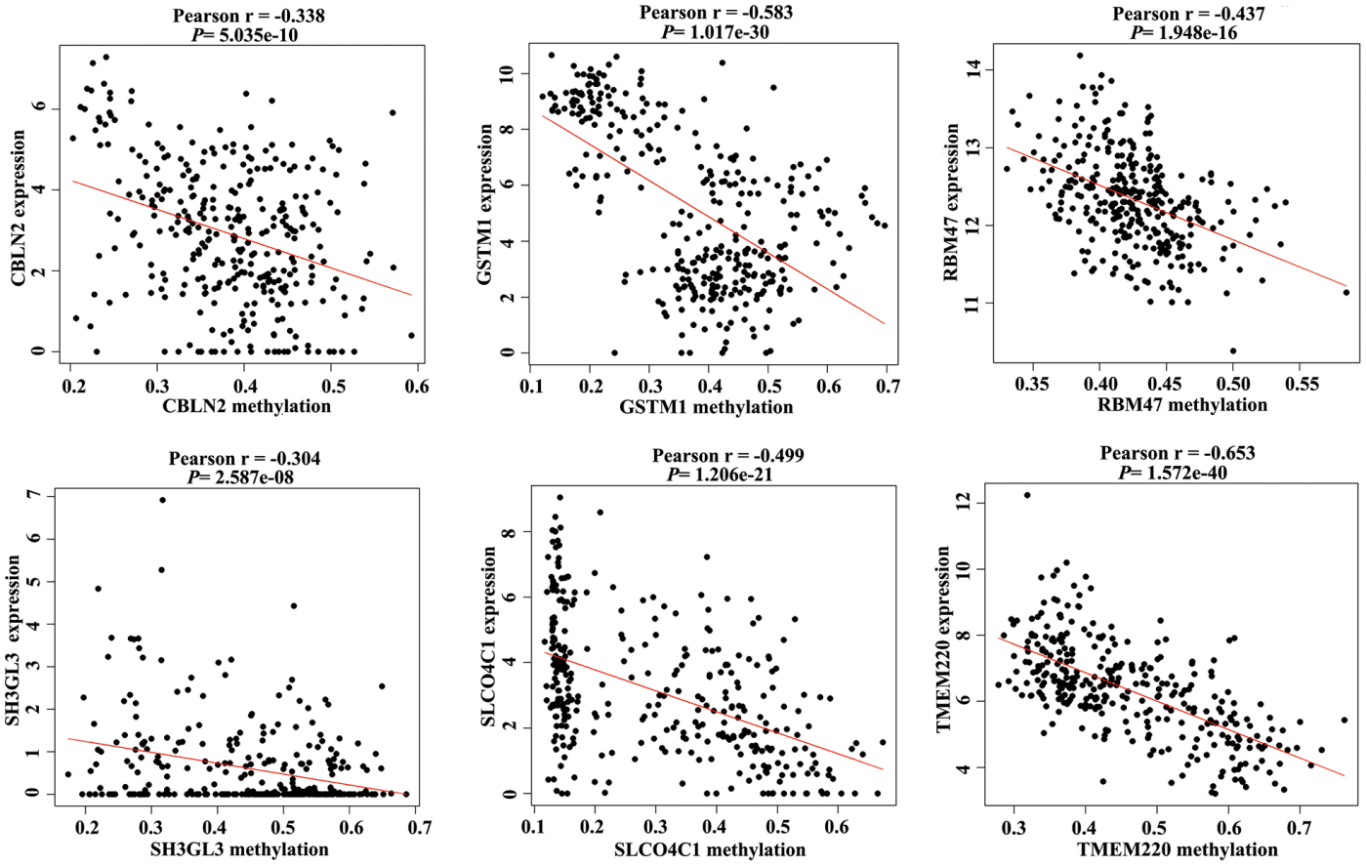
Correlation between the expression value and methylation value of the methylation-driven genes (MDGs) in colon adenocarcinoma (COAD) tissues.

Promoter hypermethylation can trigger transcriptional silencing of cancer-related genes. Therefore, we selected genes with high methylation and low expression for further analysis. We evaluated the Pearson coefficients from gene expression and methylation values for aberrantly methylated DEGs. In total, 97 aberrantly methylated DEGs were identified as MDGs (Pearson coefficient < -0.3 and *P* < 0.05; Figure 2 and Supplementary Table 1).

### Development of a risk prediction model of COAD patients

**Figure 3:**
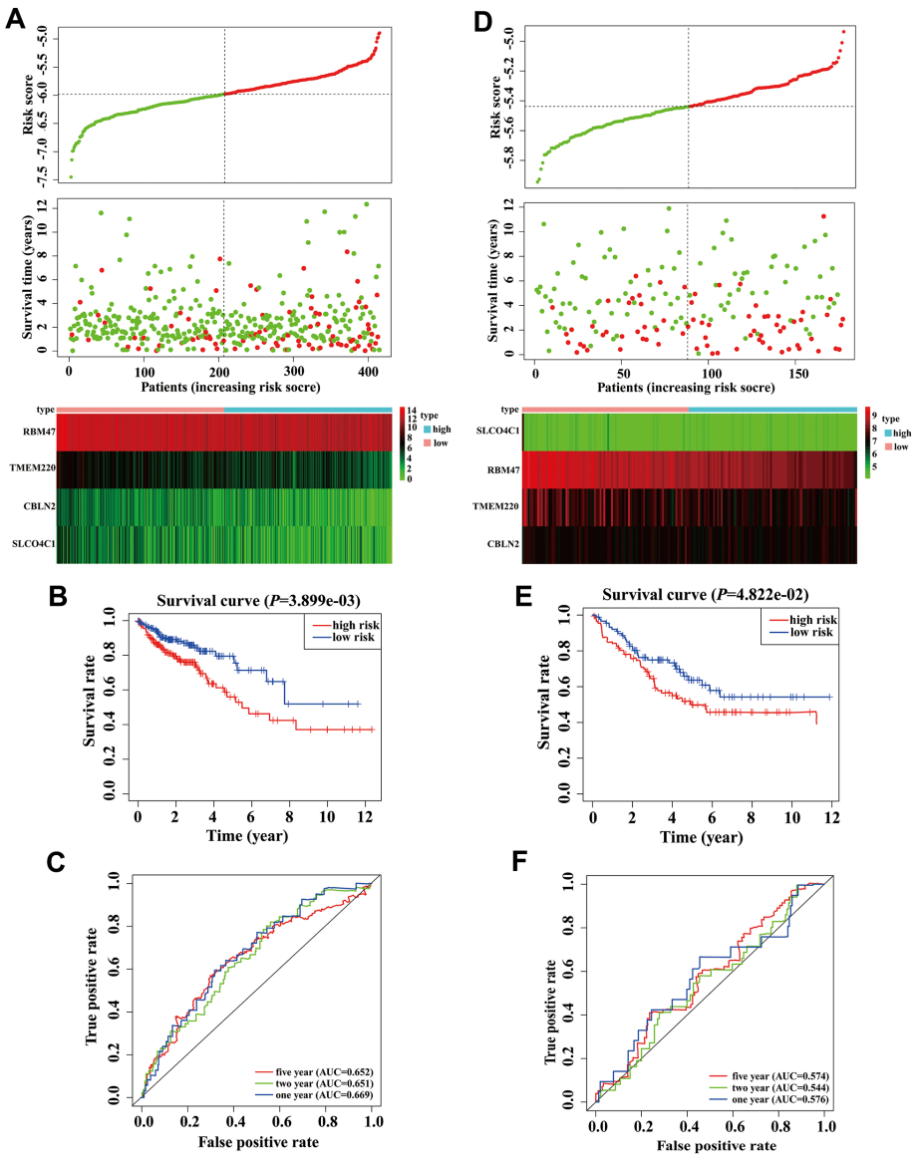
**Validation and development of risk prediction model in colon adenocarcinoma (COAD) patients.** (**A**) Risk score distribution in COAD patients, survival status of COAD patients, and expression heatmap of four methylation-driven genes (MDGs) in a Cancer Genome Atlas (TCGA) training cohort. (**B**) The K-M curve of overall survival (OS) for COAD patients between two different groups in our TCGA training cohort. (**C**) Time-dependent ROC curves at 1 year, 2 years, and 5 years in the TCGA training cohort. (**D**) Risk score distribution of COAD patients, survival status of COAD patients, and expression heatmap of four MDGs in a validation cohort. (**E**) The K-M curve of OS for COAD patients between two different groups in a validation cohort. (**F**) Time-dependent ROC curves at 1 year, 2 years, and 5 years in a validation cohort.

There were 414 COAD patients with both expression data and complete clinical information in the TCGA database, thus, we used these datasets to identify prognostic genes for COAD. Univariate Cox regression analysis initially identified that among 97 MDGs, 6 MDGs (*CBLN2*, *GSTM1*, *RBM47*, *SH3GL3*, *SLCO4C1*, and *TMEM220*) were significantly correlated with OS of COAD patients (Table 1, *P* < 0.05). *GSTM1* and *SH3GL3* were excluded due to having a Hazard ratio (HR) >1. These four prognostic genes were then utilized to build a best-fit risk prediction model using least absolute shrinkage and selection operator (LASSO) Cox regression analysis. The risk prediction formula was as follows: Risk score = (-0.121 * Expression level of *CBLN2*) + (-0.377 * Expression level of *RBM47*) + (-0.065* Expression level of *SLCO4C1*) + (-0.136 * Expression level of *TMEM220*). We then calculated the risk scores of 414 COAD patients using the formula above. The distribution of risk scores and the patients’ survival status are shown in Figure 3A. A risk heatmap was used to visualize the expression profiles of these four prognostic genes (Figure 3A). The median risk (-5.979) was used as a cutoff point to divide COAD patients into a high-risk group (n = 207) and a low-risk group (n = 207). Kaplan-Meier (K-M) analysis showed the patients in the high-risk group had worse prognosis than those in the low-risk group. (Figure 3B, *P* = 0.004). The areas under the curves (AUCs) of the 1-, 2-, and 5-year OS predictions were 0.669, 0.651 and 0.652, respectively (Figure 3C). Meanwhile, compared with any single mRNA, the signature from all four genes had higher accuracy for predicting a patients' OS (Supplementary Figure 1). These results showed that this genetic signature was effective for OS prediction.

**Table 1:** Six MDGs associated with overall survival (OS) of colon adenocarcinoma (COAD) patients.

**Gene name**	**Gene name**	**HR**	***P* value**
CBLN2	cerebellin 2 precursor	0.845	0.02
GSTM1	Glutathione S-Transferase Mu 1	1.121	0.004
RBM47	RNA Binding Motif Protein 47	0.649	0.048
SH3GL3	SH3 Domain Containing GRB2	1.278	0.032
	Like 3, Endophilin A3 solute		
SLCO4C1	carrier organic anion	0.869	0.039
	transporter family member 4C1		
TMEM220	transmembrane protein 220	0.812	0.017

In order to clarify the importance of the four prognostic genes above in COAD patients, we used the GSE17536 array data as an independent validation set. We calculated the risk scores of all patients according to the risk score formula mentioned above. As expected, scatter plots, risk heatmaps and K-M curves were able to accurately distinguish high- and low-risk group patients using this dataset (Figure 3D, 3E). The AUCs of the 1-, 2-, and 5-year OS predictions were 0.576, 0.544, and 0.574 using the GSE17536 array data, respectively (Figure 3F). These results indicated that our model was effective for OS prediction in COAD patients.

### Stratification analysis

**Figure 4:**
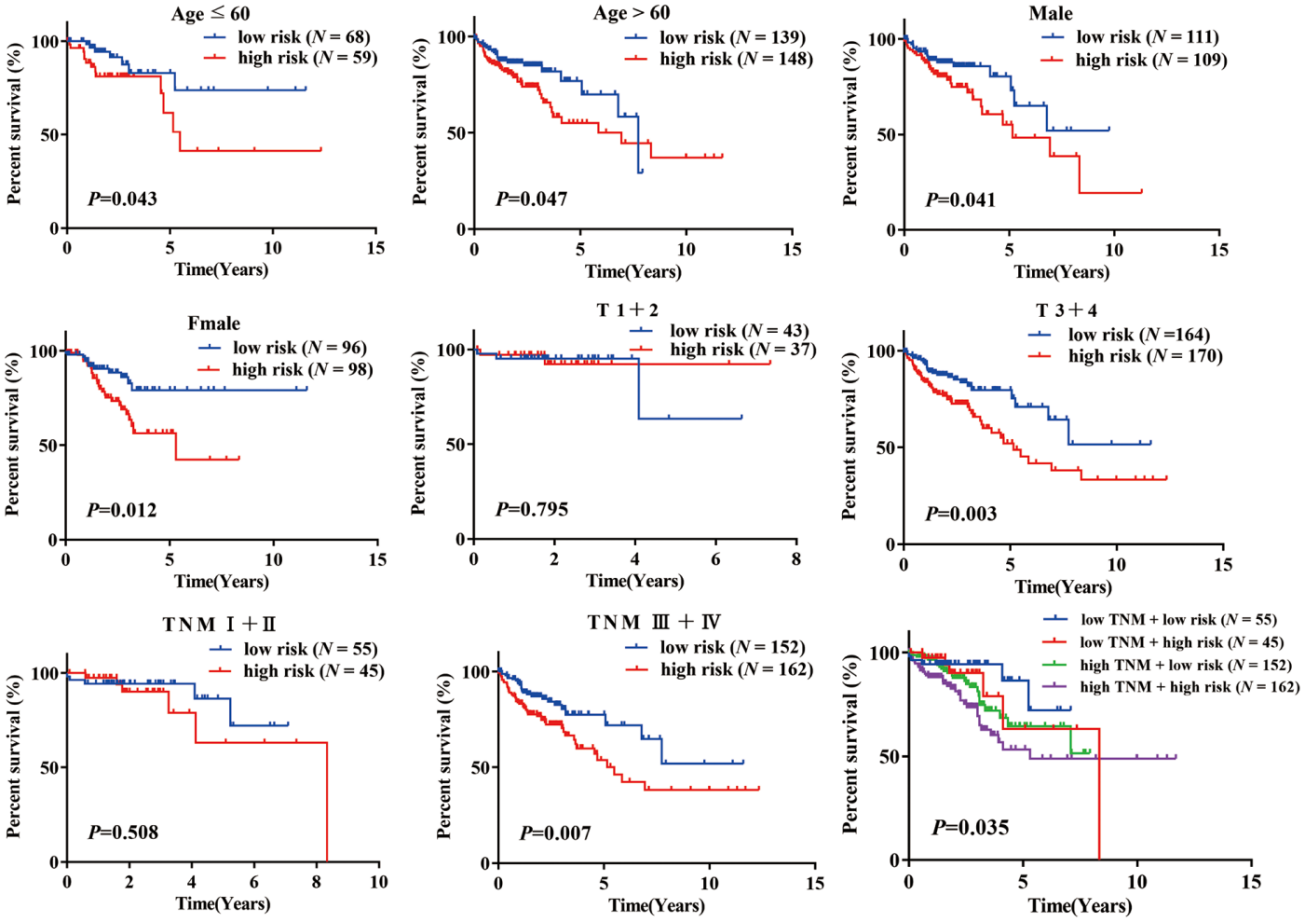
K-M analysis of overall survival (OS) for patients stratified by age, gender, T stage, and TNM stage.

A stratification analysis was then performed to investigate the applicable population using this risk prediction model and TCGA data. Patients were assigned to different subgroups based on their age (≤ 60 / > 60), sex (female/male), T stage (1 + 2/ 3 + 4) and TNM stage (I + II / III + IV). The results showed that our risk prediction model could delineate high- and low-risk patients in each age, sex, T staging, and TNM staging subgroup. Unfortunately, the risk prediction model lost its prognostic value in patients of grade T (1 + 2) and TNM (I + II), which we attributed to the small sample size of these subgroups in the TCGA COAD dataset (Figure 4).

### Establishment and evaluation of a predictive nomogram

**Figure 5:**
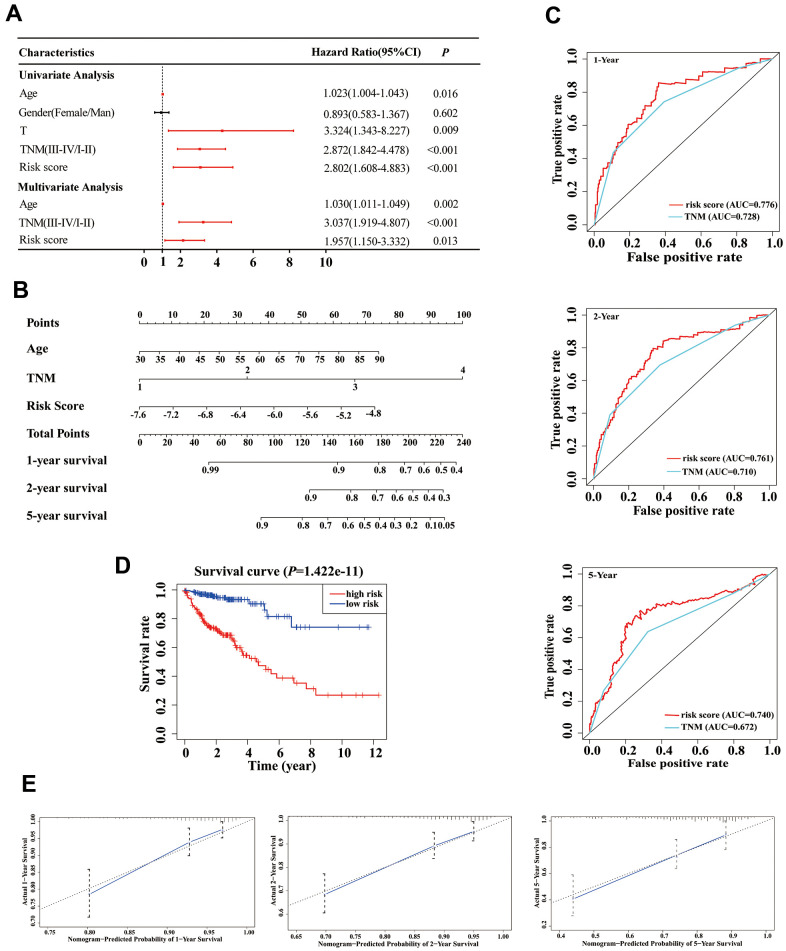
**Establishment of an overall survival (OS) nomogram for colon adenocarcinoma (COAD) patients.** (**A**) Univariate and multivariate analyses of risk score and clinical variables. Red solid dots represent significant difference, and black solid dots mean no difference. (**B**) A nomogram individually predicting OS in COAD patients. (**C**) The time-dependent ROC of our nomogram and TNM stage in the prediction of prognosis at 1-, 2-, and 5-year time points. (**D**) The K-M curve of our nomogram. (**E**) Calibration plot of the nomogram. The predicted and the actual probabilities of OS are plotted using blue solid and black dotted lines, respectively.

Based on our stepwise and multivariate Cox regression analyses, we found that age, TNM stage and risk score were independent prognostic factors in 414 COAD patients (Figure 5A). Considering the different clinical characteristics of each patient, we built a nomogram that combined age, TNM stage, and risk score to individually predict the 1-, 2-, and 5-year OS of COAD patients (Figure 5B). The AUCs of the 1-, 2-, and 5-year of this nomogram were 0.776, 0.761 and 0.740, respectively (Figure 5C). The AUCs of 1-, 2-, and 5-year prognosis from traditional TNM stage were 0.728, 0.710, and 0.672, respectively (Figure 5C). At the same time, the concordance index (C-index) of the nomogram was significantly higher than traditional TNM stage (0.755 versus 0.706, *P* < 0.05). Therefore, in terms of predicting the OS of COAD patients, our nomogram was better than traditional TNM staging. Based on the median of the nomogram score as a cutoff value, patients were then divided into high-risk and low-risk groups. K-M analysis revealed that the high-risk group had significantly poorer OS (Figure 5D, *P* < 0.001). The calibration curves of our nomogram suggested that the predicted OS was consistent with the observed OS (Figure 5E).

**Figure 6:**
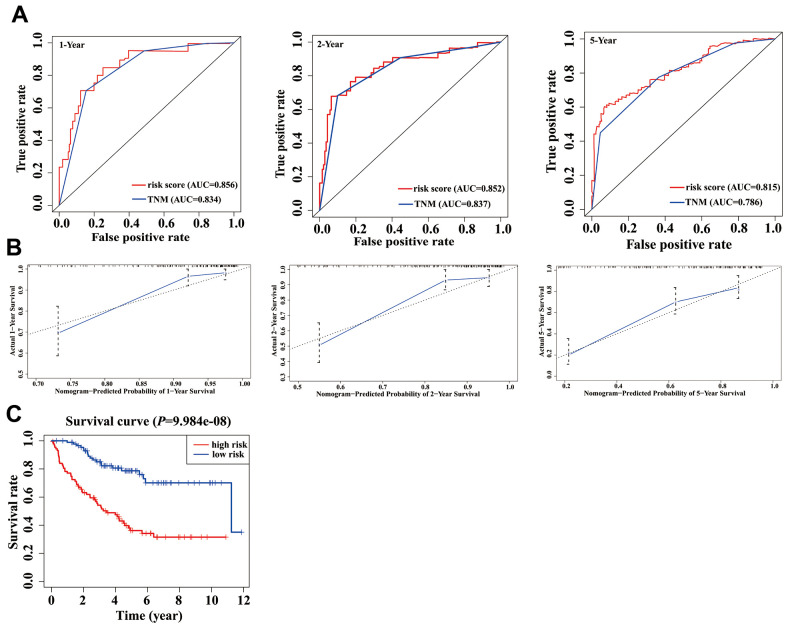
**Validation of nomogram in a validation cohort.** (**A**) Shown is the time-dependent ROC curves for 1-, 2-, and 5-year overall survival (OS) predictions from our nomogram compared with TNM stage. (**B**) Calibration curve for our nomogram in a validation cohort. The predicted and the actual probabilities of OS are plotted using blue solid and black dotted lines, respectively. (**C**) OS of our nomogram in a validation cohort.

In the validation phase, a new nomogram still showed a higher predictive efficacy in using GSE17536 array. Similar to its performance in the TCGA cohort, the AUCs of the 1-, 2-, and 5-year nomograms were greater than those from TNM stage, respectively (Figure 6A). The calibration curves of the nomograms from 1-, 2- and 5-year OS displayed obvious concordance between the predicted OS and the observed OS, respectively (Figure 6B). In addition, the C-index values of the nomogram and TNM stage were 0.778 and 0.774, respectively. Meanwhile, K-M curves could still distinguish high - and low-risk group patients (Figure 6C).

### Encyclopedia of genes and genomes (KEGG) enrichment of four candidate genes

**Figure 7:**
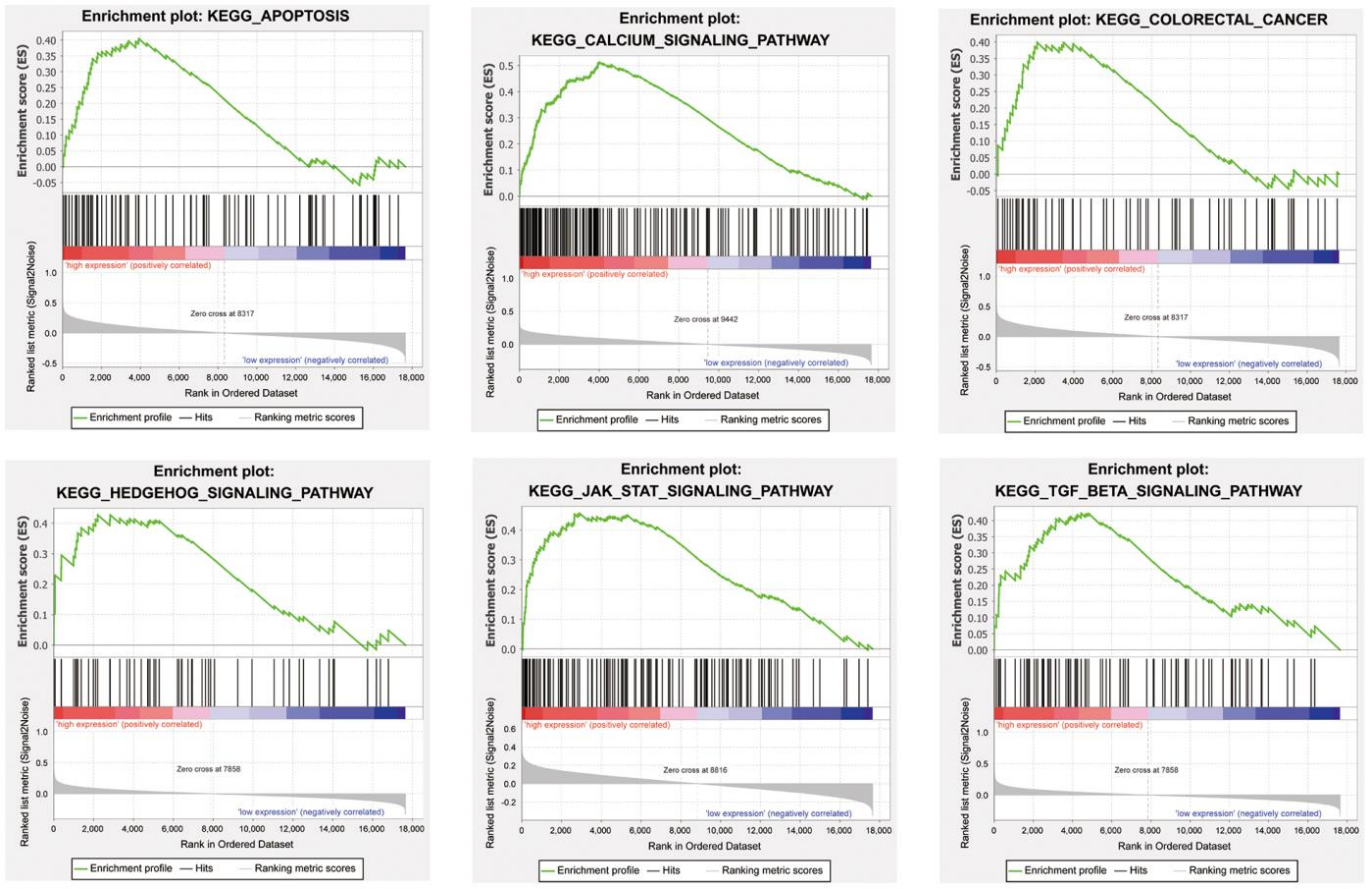
Representative enriched pathways in four candidate genes from gene set enrichment analysis (GSEA) software.

We performed gene set enrichment analysis (GSEA) with our four candidate genes to investigate the potential biological mechanisms via these genes in COAD progression. Patients were divided into high-expression and low-expression groups based on the median expression value of these candidate genes. The results showed that the four candidate genes were involved in multiple tumor-associated pathways, such as the apoptosis, the calcium signaling pathway, the colorectal cancer, the Hedgehog signaling pathway, the JAK-STAT signaling pathway, and the TGF-β signaling pathway (Figure 7, Supplementary Table 2–5).

### Validation of differential expression of *CBLN2* and *TMEM220* due to promoter methylation

**Figure 8:**
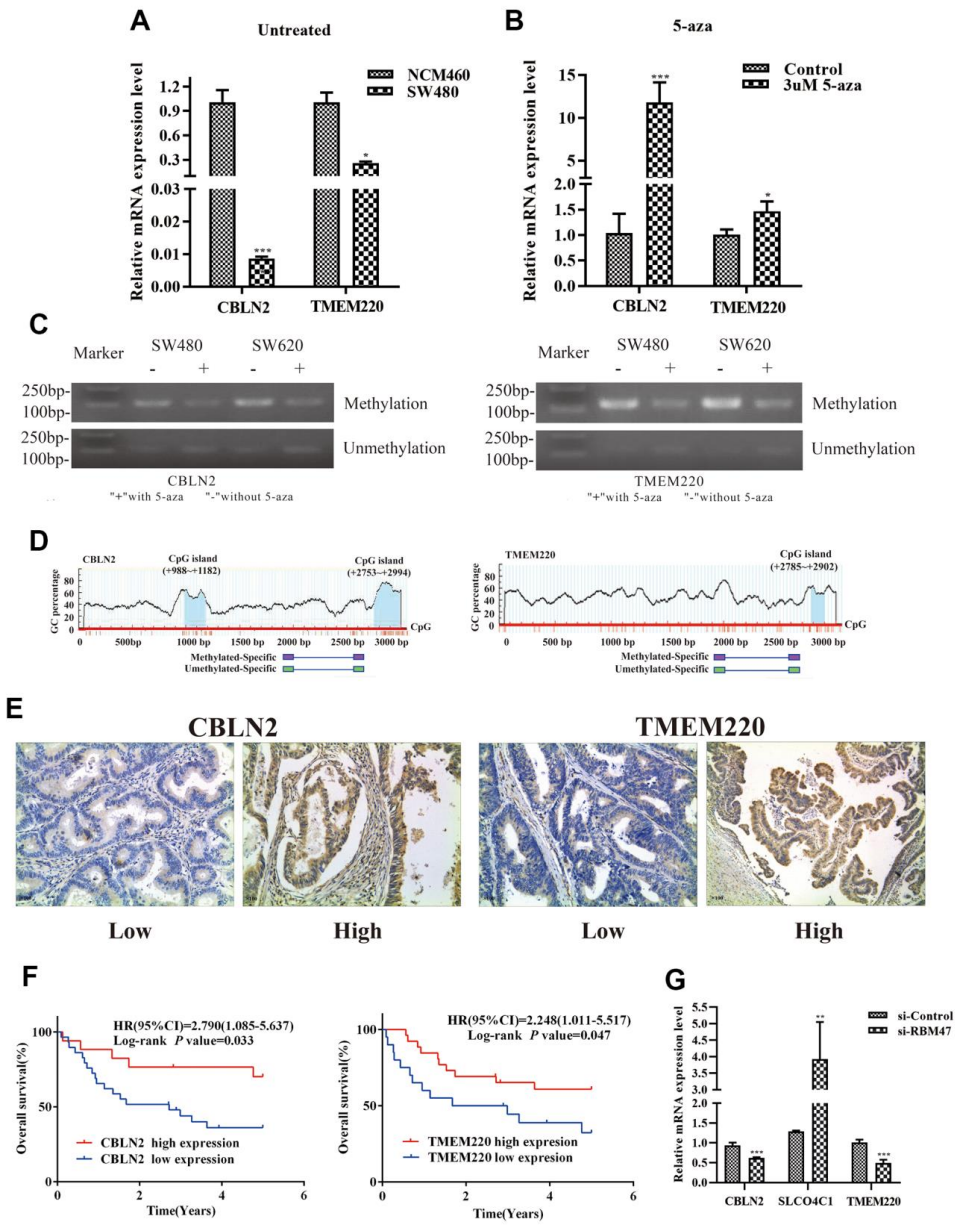
**Experimental verification in colon cells and tissues.** (**A**) qPCR was performed to identify the relative expression of *CBLN2* and *TMEM220* in NCM460 and SW480 cells. (**B**) qPCR was carried out to assess *CBLN2* and *TMEM220* expression levels in SW480 cells before and after treatment with 5-aza. (**C**) Methylation status of *CBLN2* and *TMEM220* was determined by MSP in SW480 and SW620 cells. (**D**) Schematic diagrams of CpG islands in the promoter regions of *CBLN2* and *TMEM220*. (**E**) Representative images of immunohistochemistry staining of colon sections from colon adenocarcinoma (COAD) tissues (n = 46). Original magnification, ×100. (**F**) Prognostic significance of *CBLN2* and *TMEM220* expression in COAD patients. (**G**) Knockdown of RBM47 gene in sw480 cells.

First, to further verify our results based on data from the TCGA and GEO databases, we used quantitative real-time PCR (qPCR) to determine candidate gene expression levels in NCM460 cells and SW480 cells, respectively. We found that the expression of *CBLN2* and *TMEM220* was low in SW480 cells, but very high in NCM460 cells (Figure 8A). Second, in order to determine whether abnormally methylated promoter regions directly caused transcriptional silencing of *CBLN2* and *TMEM220*, SW480 cells were treated with the DNA methyltransferase inhibitor 5-Aza-2′-deoxycytidine (5-aza), and the expression of *CBLN2* and *TMEM220* was determined via qPCR. This study found that the expression of *CBLN2* and *TMEM220* was restored in SW480 cells after treatment with 5-aza (Figure 8B). Third, methylation-specific PCR (MSP) was applied to identify the methylation status of the *CBLN2* and *TMEM220* promoter regions. Studies have previously shown that these regions are partially methylated in SW480 and SW620 cells (Figure 8C). CpG islands situated in the *CBLN2* and *TMEM220* promoter regions and the designed MSP primers are shown in Figure 8D. In summary, we confirmed that the expression of *CBLN2* and *TMEM220* was silenced by the methylation of these promoter regions in a COAD cell line.

### Relationship between *CBLN2* and *TMEM220* expression and OS of COAD patients

The expression of *CBLN2* and *TMEM220* in 46 COAD tissues was then examined by immunohistochemistry. *CBLN2* and *TMEM220* protein expression levels were significantly different in tumor tissues as compared to controls (Figure 8E). A marker was considered positive when 20% or more cells were stained [17]. The prognostic effects of *CBLN2* and *TMEM220* on the OS of COAD patients were next evaluated through K-M analysis. As shown in Figure 8F, both *CBLN2* and *TMEM220* led to significant survival differences in 46 COAD samples (*P* = 0.033 and *P*=0.047, respectively). The clinical information for these patients is listed in Table 2.

**Table 2:** Clinical information of the study patients.

**Characteristics**	**Number (*N* = 46)**
Age (years)	60.61±9.97
Sex	
Male	29(63.04%)
Female	17(36.96%)
Smoking	
Yes	19(41.30%)
No	27(58.70%)
Drinking	
Yes	18(39.13%)
No	28(60.87%)
Tumor size (cm)	
< 5×4	19(41.30%)
≥ 5×4	18(39.13%)
Unknown	9(19.57%)
TNM stage	
I	5(10.87%)
II	13(28.26%)
III	12(26.09%)
IV	13(28.26%)
Unknown	3(6.52%)

### Effects of *RBM47* gene knock-out on *CBLN2*, *SLCO4C1* or *TMEM220* expression levels

Considering that *RBM47* had the greatest influence on our risk prediction model, we further explored the correlation between *RBM47* and *CBLN2*, *SLCO4C1* and *TMEM220*. The expression values of *CBLN2* and *TMEM220* were decreased and the expression value of *SLCO4C1* was increased in SW480 cells when *RBM47* was knocked out (Figure 8G).

## DISCUSSION

COAD is a fatal malignancy, mainly caused by malignant transformation of colon epithelial cells [18, 19]. Despite surgical resection with curative intent often being performed to treat COAD, the clinical outcome of patients with COAD remains poor [20, 21]. As a result of multi-Omics data and analysis, there has been a growing recognition that COAD is a molecularly heterogeneous disease [22, 23]. Recently, studies have started to emphasize genome-wide changes in expression and epigenetics as they relate to COAD, as well as evaluating their interactions to provide a more complete molecular profile of this disease [24–26]. In the present study, a joint analysis of clinical data and multiscale omics data was utilized to investigate the epigenetic changes that may drive the initiation and progression of COAD. Simultaneously, we identified a powerful DNA methylation signature and nomogram for prognosis prediction of COAD in patients.

Abnormal DNA methylation patterns occur frequently in tumors. Among these dysregulated genes driven by DNA methylation, some may promote malignant transformation via overexpression of oncogenes or knockdown of tumor suppressor genes (TSGs), which leads to the disorder of the tumor microenvironment and may be a prognostic biomarkers for tumors [27, 28]. In this study, we identified 659 abnormally methylated DEGs by comprehensive analysis of DNA methylation and transcriptome data from the TCGA and GEO databases. Simultaneously, we calculated the Pearson coefficient between the expression and methylation values of 659 abnormal methylated DEGs, yielding a total of 97 MDGs. Using a univariate Cox regression model, we determined that four MDGs (*CBLN2*, *RBM47*, *SLCO4C1*, and *TMEM220*) were protective genes for prognosis in COAD patients (HR < 1). While the efficacy of any single marker is often limited, a multi-marker signature can have greater diagnostic and prognostic value [29]. Thus, we constructed a risk prediction model based on these four MDGs, which had a high value in predicting the prognosis of COAD patients. The survival curves showed that the prognosis of patients in the low-risk group were significantly better than those in the high-risk group. A time-dependent receiver operating characteristic (ROC) curve confirmed that there was higher prediction accuracy when predicting the OS at 1, 2, and 5 years. Stratification analyses show that this model was widely applicable in populations with different clinicopathologic features. In order to facilitate the personalized prediction of the OS of COAD patients, we combined age, TNM stage and risk score to construct a nomogram. This nomogram had excellent performance when used to predict the OS of COAD patients. In fact, compared with the traditional TNM staging system, our nomogram provided higher accuracy for prognosis of COAD patients. In order to test the issue of overfitting of our risk prediction model and nomogram, we used the GSE17536 external independent array to verify these two new models, and found that they still had high predictive performance in the OS of COAD patients.

Our risk prediction model consisted of four gene members, some of which have been reported to be regulated by DNA methylation in cancer and other diseases. *RBM47* was previously described to act as a tumor-suppressive role in colorectal and breast cancer, and low *RBM47* expression was significantly associated with poor OS in COAD and CRC patient cohorts [30, 31]. Meanwhile, compared with prediction using *RBM47* alone, we also found that a multi-marker signature could improve the diagnostic and prognostic value in COAD patients. Rokavec et al. found that *RBM47* protein expression was higher in normal colonic mucosa than in adjacent tumor tissue in the majority of cases [30]. The hypermethylation of the promoter of *RBM47* had been detected in nonfunctioning pancreatic neuroendocrine tumors [32]. We also found that *CBLN2* and *TMEM220* expression were down-regulated and *SLCO4C1* expression was up-regulated in SW480 cells with *RBM47* knockdown, which suggested tha*t CBLN2*, *SLCO4C1*, and *TMEM220* were involved in the development of COAD under the regulation of *RBM47*. A large number of previous studies have shown that mRNA expression levels are regulated by promoter methylation of *SLCO4C1* in cancers, such as colorectal cancer [33], prostate cancer [34] and head and neck cancers [35]. However, as the relationship between the expression of *CBLN2* and *TMEM220* and DNA methylation transcriptional silencing has not been previously reported in COAD, we conducted qPCR and MSP analysis in NCM460, SW480, and SW620 cells, and found a DNA methylation transcriptional silencing relationship for *CBLN2* and *TMEM220*. Moreover, the low expression of *CBLN2* and *TMEM220* was associated with poor prognosis in COAD patients by immunohistochemistry. Buffart et al. found that the *TMEM220* mRNA expression level in gastric cancer was regulated by the methylation status in the promoter region [36]. Wang et al. found significant mutations in *CBLN2* in patients with esophageal small cell carcinoma [37], but its role in tumors has not been revealed yet.

Although the performance of our risk prediction model and nomogram was quite favorable, our study still had limitations. First, the sample size in our verification set was not large enough. Therefore, in the future, it will be necessary to use an external data set with a large sample size comprising complete clinical information and multi-omics information for verification. Second, our experimental data was inadequate, and lacked some verification information on the differences in the expression and methylation of our four MDGs in COAD tissues.

## MATERIALS AND METHODS

### Materials acquisition and preprocessing

DNA methylation data, transcriptome data and corresponding clinical data about COAD tissues were obtained from the TCGA (https://portal.gdc.cancer.gov/) and GEO (https://www.ncbi.nlm.nih.gov/geo/) databases in April, 2019. Gene methylation data from the TCGA dataset was generated using the Illumina Infinium HumanMethylation450 microarray, which included 310 COAD and 37 adjacent non-tumor samples. If any gene had multiple cg sites, the empty sites were removed and the mean value of β was used to represent its methylation level [38]. Gene transcriptome data from the TCGA database (Level 3) was normalized and log2 scaled using the functions DEGList and calcNormFactors in the edgeR package for R [39], which included 473 COAD and 41 adjacent non-tumor samples. Gene methylation data from the GSE48684 arrays was generated using the GPL13534 platform (Illumina HumanMethylation450 BeadChip). Gene transcriptome data from the GSE39582 and GSE17536 arrays was generated using the GPL570 platform (Affymetrix Human Genome U133 plus 2.0 Array). The GSE48684 array consisted of 106 COAD and 41 adjacent non-tumor samples. The GSE39582 array consisted of 566 COAD and 19 adjacent non-tumor samples. The GSE17536 array consisted of 177 COAD samples. We also retrospectively collected 46 cases of COAD tissues from patients who underwent surgical resection in the Fourth Hospital of Hebei Medical University, China (from December 2010 to December 2013). All patients had resectable COAD, and none of them had received preoperative anticancer treatments. They were followed until June 2018. Ethical permission of this study protocol was granted by the ethical committee of Hebei Medical University. All patients were informed and signed informed consent forms prior to enrollment in the study.

### Identification of aberrantly methylated DEGs

For TCGA transcriptome data, the EdgeR package was used to identify the DEGs between COAD and non-tumor samples, and an absolute value of the log2 fold change (|log_2_FC|) >1 and false discovery rate (FDR) < 0.05 were considered statistically significant. For GEO transcriptome data, the limma package was used to identify DEGs between COAD and non-tumor samples, with the thresholds of FDR < 0.01 and |log_2_FC| > 0.5. All methylation data was analyzed with the limma package. Herein, genes with FDR < 0.05 were considered as DMGs. Finally, aberrantly methylated DEGs were detected by overlapping DEGs and DMGs in Venny software 2.1 (http://bioinfogp.cnb.csic.es/tools/venny/).

### Correlation analysis of aberrantly methylated DEGs

To study the transcriptional regulation of DNA methylation, we evaluated the Pearson coefficient between gene expression and the methylation data for aberrantly methylated DEGs. A total of 322 COAD samples with matching methylation data and expression data were used for correlation analysis. Aberrantly methylated DEGs with a Pearson coefficient < -0.3 and *P* < 0.05 were defined as MDGs [40]. Scatter plot of these MDGs was plotted using ggplot2 in R.

### Development of a risk prediction model

Initially, univariate Cox regression analysis was used to evaluate the association between MDGs and the OS of COAD patients, and MDGs with a *P* < 0.05 were selected for further analysis. Based on the expression value of MDGs, the LASSO Cox regression models were used to develop a best-fit risk prediction model with the R package “glmnet”. The risk score for each COAD patient was calculated as follows:

$$\text{Risk}\;\text{score}=\sum_{i=1}^{n}\exp_{\text{i}}{}^{*}\beta \text{i},$$

where n is the number of prognostic genes, exp_i_ is the expression value of each gene i, and βi is the weighted regression coefficient in gene i from multivariate Cox regression analysis. Then, time-dependent ROC curve and K-M analyses were used to evaluate the predictive ability of our model. In the validation phase, we verified the risk prediction model using the GSE17536 dataset, another COAD cohort.

### Construction and assessment of nomograms

Stepwise and multivariate Cox proportional hazard regression models were used to distinguish independent prognostic parameters of COAD patients, based on which we developed a nomogram. K-M analysis, time-dependent ROC curve analysis, calibration plot and C-indices were used to evaluate the discriminative ability of our nomogram. A C-index was calculated to assess nomogram discrimination by means of the bootstrap method with 1000 resamples. We assessed the performance of our nomogram on predicting OS for COAD patients using traditional TNM stage as a control. Meanwhile, the GSE17536 cohort was used to verify the new nomogram.

### Pathway enrichment analysis of MDGs

GSEA was carried out to explore the underlying biological mechanisms of each marker. GSEA software was downloaded from the GSEA home (http://software.broadinstitute.org/gsea/index.jsp). “c2.cp.kegg.v7.2.symbols.gmt gene sets” was used as a reference gene set to enrich KEGG pathways for candidate genes in TCGA. Last, |NES| > 1 and *P* < 0.05 were set as thresholds.

### Validation experiments in colonic cells

To verify the transcriptional silencing relationship of prognostic genes, we used three human colon cell lines (NCM460, SW480, and SW620) for validation. NCM460 and SW480 cells were cultured in RPMI 1640 (Gibco, Carlsbad, CA, USA). SW620 cells were cultured in DMEM (Gibco, Shanghai, China). All cell culture medium was supplemented with 10% fetal bovine serum (Invitrogen, Carlsbad, CA, USA) and 1% penicillin/streptomycin. To investigate the effect of 5-aza (Sigma, St. Louis, MO, USA) treatment, SW480 cells were treated with 3 μM for 72 h [41]. Meanwhile, SW480 cells were transfected with control and *RBM47* siRNAs (Thermo Fisher, USA) according to the manufacturer's protocol. Cells were siRNAs were treated for 48 h and then switched to media lacking siRNA. Total RNA was then isolated from cells utilizing the Trizol method (Invitrogen, Shanghai, China). qPCR was performed on an ABI 7500 real-time PCR System (Applied Biosystems, Carlsbad, CA) using SYBR Green (Takara, Japan). GAPDH was used as an internal reference, and the relative expression level of each gene of interest was calculated with the formula 2^-ΔΔCt^ [42]. The methylation status of MDGs was tested in SW480 and SW620 cells by MSP. We predicted CpG islands and designed MSP primers with Methyl Primer Express software v1.0 (Thermo Fisher Scientific, Waltham, MA) based on the genomic sequence around the transcriptional start site (TSS) of each gene. qPCR and MSP primers are illustrated in Supplementary Table 6.

### Immunohistochemistry

Paraffin-embedded specimens from colon tissues were sectioned to a 5 μm thickness. The sections were then deparaffinized in xylene and rehydrated through graded alcohol solutions. Antigen extraction was performed using citrate buffer (pH 6.0), and sections were stored in Tris buffered saline (TBS). Endogenous peroxidase activity was blocked by incubation in 3% hydrogen peroxide. The sections were incubated with the anti-CBLN2 antibody (1:100, Abcam, Shanghai, China) or anti-TMEM220 antibody (1:100, Abcam, Shanghai, China) overnight at 4° C. The reaction products were visualized with diaminobenzidine (Vector labs, Burlingame, CA, USA) as the chromogen and counterstained with hematoxylin. Finally, images were acquired with immunofluorescence microscopy.

### Statistical analysis

All statistical analysis was performed in R 3.5.0 and GraphPad Prism 6.0 (GraphPad Software, La Jolla, CA, USA). A two-sample *t*-test was used to compare gene expression levels in colon cell lines. *P* < 0.05 was considered statistically significant.

## Supplementary Material

Supplementary Figure 1

Supplementary Table 1

Supplementary Tables 2, 3, 4, 5 and 6
