# Supplementary material for: Development and validation of a risk prediction model and nomogram for colon adenocarcinoma based on methylation-driven genes
Source: Aging (Albany NY). 2021 Jun 28;13(12):16600–19. doi: 10.18632/aging.203179 (PMC8266312; doi:10.18632/aging.203179)
Supplement: Supplementary Figure 1 [file aging-13-203179-s001.pdf]

SUPPLEMENTARY FIGURE

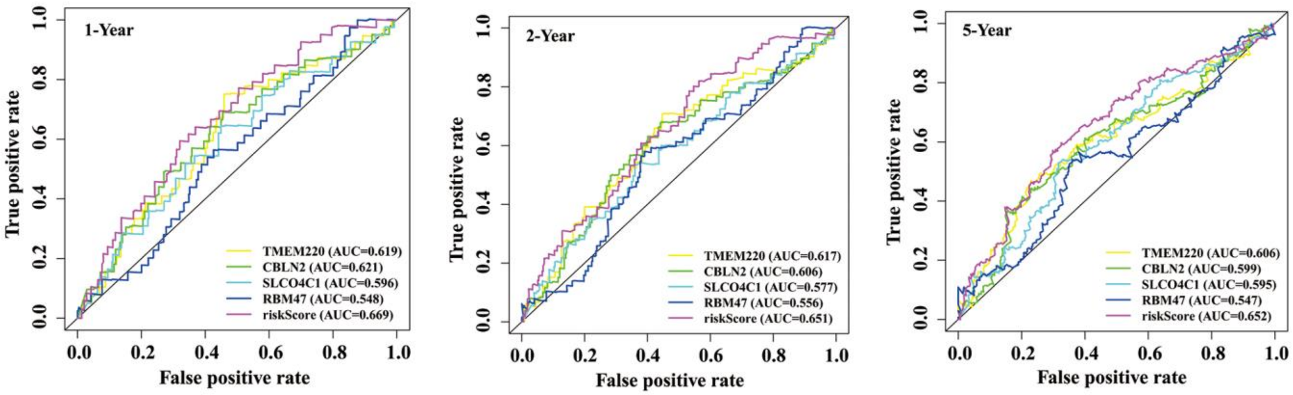

Supplementary Figure 1. Comparison of prognostic accuracy between 4 gene signature and single mRNAs.
