# Supplementary material for: Development and validation of a risk prediction model and nomogram for colon adenocarcinoma based on methylation-driven genes
Source: Aging (Albany NY). 2021 Jun 28;13(12):16600–19. doi: 10.18632/aging.203179 (PMC8266312; doi:10.18632/aging.203179)
Supplement: Supplementary Table 1 [file aging-13-203179-s002.doc]

| **Supplementary Table 1. A total of 97 MDGs were screened in COAD patients from TCGA data** | | | | | | | | | | | | | | |
| --- | --- | --- | --- | --- | --- | --- | --- | --- | --- | --- | --- | --- | --- | --- |
| **Gene symbol** | **Methylation** | | | | |  | **Expression** | | | | |  | **Correlation analysis** | |
|  | **GSE48684** | |  | **TCGA** | |  | **GSE39582** | |  | **TCGA** | |  | **TCGA** | |
|  | **Log_2_ FC** | **FDR** |  | **Log_2_ FC** | **FDR** |  | **Log_2_ FC** | **FDR** |  | **Log_2_ FC** | **FDR** |  | **Pearson r** | ***P*** |
| CES3 | 0.0649642 | 0.0311602 |  | 0.1806103 | 0.0012 |  | -2.232786 | 1.422E-35 |  | -2.104215 | 2.234E-34 |  | -0.72 | <0.05 |
| PIGR | 0.2222292 | 1.132E-06 |  | 0.4062354 | 2.7E-10 |  | -3.43206 | 9.787E-13 |  | -1.577659 | 2.264E-09 |  | -0.719 | <0.05 |
| VSIG2 | 0.1535406 | 4.803E-07 |  | 0.3466555 | 9E-13 |  | -3.24417 | 7.884E-34 |  | -2.699208 | 2.216E-26 |  | -0.705 | <0.05 |
| GGT6 | 0.1413684 | 0.0012741 |  | 0.1687376 | 0.00102 |  | -2.216477 | 2.701E-14 |  | -1.699196 | 3.294E-22 |  | -0.675 | <0.05 |
| KLHL34 | 0.3302537 | 3.33E-06 |  | 0.4611482 | 5.5E-10 |  | -0.648202 | 1.227E-21 |  | -2.015343 | 4.592E-11 |  | -0.663 | <0.05 |
| TMEM220 | 0.1657459 | 0.0079166 |  | 0.3518165 | 4.3E-08 |  | -2.708836 | 1.872E-38 |  | -2.918586 | 7.636E-97 |  | -0.653 | <0.05 |
| CES2 | 0.0974754 | 0.00155 |  | 0.0779113 | 0.00954 |  | -2.083763 | 5.926E-14 |  | -2.041185 | 3.726E-43 |  | -0.631 | <0.05 |
| TMEM35 | 0.203501 | 1.186E-06 |  | 0.2966535 | 8.3E-13 |  | -1.187033 | 2.93E-07 |  | -3.918953 | 1.244E-90 |  | -0.612 | <0.05 |
| CD52 | 0.0679024 | 0.0105825 |  | 0.0825758 | 0.00143 |  | -0.827953 | 0.0007644 |  | -1.175308 | 1.574E-12 |  | -0.595 | <0.05 |
| FOXD2 | 0.4442766 | 3.23E-09 |  | 0.8707129 | 1.2E-15 |  | -1.042362 | 1.634E-13 |  | -1.067522 | 6.034E-12 |  | -0.593 | <0.05 |
| GSTM5 | 0.0695634 | 0.0100393 |  | 0.1935774 | 2.5E-09 |  | -0.538855 | 1.094E-14 |  | -2.81808 | 2.793E-84 |  | -0.589 | <0.05 |
| HMGCS2 | 0.0985826 | 0.0068679 |  | 0.116338 | 0.01833 |  | -2.80666 | 2.362E-14 |  | -2.235434 | 3.628E-19 |  | -0.588 | <0.05 |
| GSTM1 | 0.2998824 | 0.0025126 |  | 0.4539683 | 2.3E-06 |  | -0.61584 | 1.796E-05 |  | -1.265102 | 8.773E-05 |  | -0.583 | <0.05 |
| SLC9A2 | 0.1617344 | 8.556E-05 |  | 0.3994031 | 1E-12 |  | -1.859299 | 1.789E-39 |  | -2.020061 | 7.383E-30 |  | -0.583 | <0.05 |
| ADHFE1 | 0.3559227 | 1.159E-12 |  | 0.5854648 | 4.9E-20 |  | -0.665721 | 7.576E-18 |  | -2.733025 | 7.012E-89 |  | -0.577 | <0.05 |
| B3GNT7 | 0.1420685 | 3.761E-06 |  | 0.3957301 | 2.4E-18 |  | -1.463846 | 1.626E-17 |  | -3.151146 | 8.167E-53 |  | -0.568 | <0.05 |
| ANO5 | 0.7118491 | 1.178E-07 |  | 1.0401407 | 1E-14 |  | -1.945004 | 2.178E-38 |  | -3.04212 | 3.126E-39 |  | -0.558 | <0.05 |
| CA12 | 0.029191 | 0.0499169 |  | 0.1301385 | 1E-06 |  | -2.216366 | 3.128E-22 |  | -2.18258 | 2.519E-44 |  | -0.552 | <0.05 |
| ITM2C | 0.1417016 | 1.16E-07 |  | 0.3207834 | 1.1E-17 |  | -2.522464 | 5.673E-28 |  | -2.171988 | 2.41E-57 |  | -0.545 | <0.05 |
| KLF4 | 0.1711838 | 0.012993 |  | 0.5178819 | 1.8E-08 |  | -2.853672 | 5.12E-28 |  | -2.390495 | 1.927E-76 |  | -0.542 | <0.05 |
| KLF9 | 0.1932846 | 7.601E-05 |  | 0.8225037 | 1.3E-14 |  | -0.735044 | 8.838E-14 |  | -1.699533 | 1.578E-37 |  | -0.537 | <0.05 |
| PROM2 | 0.1491484 | 2.415E-09 |  | 0.2230607 | 1.8E-18 |  | -0.529202 | 5.82E-10 |  | -1.082358 | 2.476E-06 |  | -0.522 | <0.05 |
| FBLIM1 | 0.5311242 | 2.45E-13 |  | 0.7969276 | 2.4E-19 |  | -0.950545 | 8.583E-22 |  | -1.25799 | 7.684E-62 |  | -0.515 | <0.05 |
| C2orf40 | 0.3825655 | 5.373E-12 |  | 0.5484147 | 7.2E-20 |  | -1.164801 | 3.088E-09 |  | -4.093753 | 5.799E-74 |  | -0.506 | <0.05 |
| SLCO4C1 | 0.9706848 | 2.079E-07 |  | 1.1543829 | 4.8E-10 |  | -0.620421 | 9.009E-33 |  | -3.483021 | 7.76E-56 |  | -0.499 | <0.05 |
| VLDLR | 0.1934145 | 0.0017774 |  | 0.4217926 | 1.2E-13 |  | -1.784415 | 3.816E-14 |  | -1.069368 | 5.197E-10 |  | -0.498 | <0.05 |
| BEST4 | 0.0635718 | 0.0393127 |  | 0.1569934 | 4.3E-10 |  | -3.201192 | 1.8E-120 |  | -5.82661 | 4.91E-298 |  | -0.494 | <0.05 |
| NPY | 0.6288677 | 1.071E-10 |  | 0.7730512 | 2.3E-19 |  | -0.697024 | 3.49E-15 |  | -3.442327 | 1.399E-41 |  | -0.494 | <0.05 |
| BEX4 | 0.4682415 | 2.876E-05 |  | 0.6739798 | 6.1E-07 |  | -1.285934 | 5.302E-06 |  | -1.754847 | 2.654E-26 |  | -0.475 | <0.05 |
| ACVRL1 | 0.0584008 | 0.0001409 |  | 0.0920879 | 1E-07 |  | -1.390724 | 1.344E-25 |  | -1.539204 | 5.759E-35 |  | -0.471 | <0.05 |
| RBM24 | 0.2399982 | 2.872E-06 |  | 0.4570157 | 3.2E-17 |  | -1.501965 | 1.418E-13 |  | -1.787794 | 3.469E-18 |  | -0.467 | <0.05 |
| ARMCX1 | 0.2040074 | 0.000896 |  | 0.2962822 | 5.7E-10 |  | -0.550604 | 0.0308275 |  | -1.728382 | 6.587E-34 |  | -0.464 | <0.05 |
| C10orf99 | 0.1222104 | 1.242E-06 |  | 0.117915 | 0.00016 |  | -2.983334 | 1.167E-08 |  | -1.91081 | 4.115E-16 |  | -0.46 | <0.05 |
| PTGER4 | 0.2002907 | 2.047E-06 |  | 0.3334642 | 2.4E-14 |  | -1.722505 | 4.48E-22 |  | -1.46246 | 4.542E-36 |  | -0.455 | <0.05 |
| SCNN1B | 0.2536541 | 1.557E-05 |  | 0.4125218 | 1.8E-13 |  | -3.621237 | 1.576E-52 |  | -4.153569 | 7.849E-77 |  | -0.452 | <0.05 |
| TMEM61 | 0.0743523 | 0.0077652 |  | 0.4961043 | 8E-11 |  | -0.901679 | 1.495E-07 |  | -1.317536 | 3.765E-08 |  | -0.447 | <0.05 |
| FAS | 0.0716672 | 0.0051903 |  | 0.3613489 | 2E-15 |  | -1.646711 | 1.074E-12 |  | -1.057181 | 5.035E-15 |  | -0.441 | <0.05 |
| PTGDR | 0.2312325 | 0.0001444 |  | 0.4758782 | 2.8E-11 |  | -1.449932 | 3.996E-27 |  | -1.679442 | 1.464E-08 |  | -0.441 | <0.05 |
| NAP1L2 | 0.5047095 | 0.0003741 |  | 0.852947 | 7.2E-08 |  | -1.40679 | 1.137E-43 |  | -3.508457 | 9.265E-78 |  | -0.439 | <0.05 |
| CLIP4 | 0.4889901 | 3.239E-10 |  | 0.4331696 | 2.7E-19 |  | -0.557444 | 1.85E-05 |  | -1.669456 | 1.039E-19 |  | -0.438 | <0.05 |
| RBM47 | 0.060887 | 0.0306648 |  | 0.0475145 | 0.01585 |  | -1.733487 | 3.268E-45 |  | -1.181839 | 9.929E-54 |  | -0.437 | <0.05 |
| GNA11 | 0.1220504 | 5.722E-13 |  | 0.1905177 | 4.2E-17 |  | -1.64726 | 1.216E-49 |  | -1.697253 | 6.179E-82 |  | -0.432 | <0.05 |
| MT1E | 0.327476 | 9.365E-05 |  | 0.7033285 | 1.4E-07 |  | -2.528696 | 7.956E-20 |  | -2.201041 | 1.02E-24 |  | -0.432 | <0.05 |
| FGFR2 | 0.0447884 | 0.0405098 |  | 0.0786457 | 5.2E-08 |  | -0.762787 | 9.159E-18 |  | -2.086409 | 2.697E-32 |  | -0.43 | <0.05 |
| AOX1 | 0.3920365 | 6.798E-06 |  | 0.4447178 | 9.2E-09 |  | -0.521196 | 0.0002682 |  | -1.827489 | 3.991E-21 |  | -0.413 | <0.05 |
| EPB41L3 | 0.5220754 | 3.096E-06 |  | 0.937115 | 1.6E-11 |  | -2.998555 | 2.739E-28 |  | -2.558244 | 1.725E-51 |  | -0.412 | <0.05 |
| JAM2 | 0.6486611 | 2.236E-10 |  | 1.0851459 | 3.5E-19 |  | -0.980901 | 1.262E-07 |  | -2.346934 | 6.274E-59 |  | -0.409 | <0.05 |
| B3GNT8 | 0.040017 | 0.009259 |  | 0.3483962 | 1E-10 |  | -0.873461 | 2.042E-10 |  | -1.536797 | 9.808E-28 |  | -0.406 | <0.05 |
| BMP3 | 0.6425844 | 1.23E-08 |  | 0.7983027 | 7.4E-16 |  | -1.342316 | 6.424E-62 |  | -5.222039 | 2.48E-118 |  | -0.4 | <0.05 |
| ANPEP | 0.1204134 | 1.952E-07 |  | 0.2179681 | 7.3E-15 |  | -1.136032 | 7.455E-17 |  | -3.659177 | 8.326E-50 |  | -0.398 | <0.05 |
| VILL | 0.0974366 | 0.0023611 |  | 0.1730753 | 1.1E-07 |  | -2.042015 | 1.985E-21 |  | -1.389554 | 3.828E-22 |  | -0.398 | <0.05 |
| HPSE2 | 0.1987472 | 3.146E-08 |  | 0.4815724 | 1.1E-18 |  | -0.545769 | 9.153E-11 |  | -3.184647 | 1.269E-71 |  | -0.393 | <0.05 |
| SFRP1 | 0.105485 | 1.144E-05 |  | 0.232503 | 3E-13 |  | -0.981581 | 4.883E-24 |  | -4.093528 | 4.277E-66 |  | -0.393 | <0.05 |
| SST | 0.1398723 | 0.0011936 |  | 0.2342339 | 1.3E-10 |  | -3.350982 | 1.696E-47 |  | -4.907155 | 1.027E-65 |  | -0.392 | <0.05 |
| CD1D | 0.1353711 | 0.0095904 |  | 0.4731945 | 1.9E-08 |  | -1.488516 | 3.606E-22 |  | -1.617102 | 2.689E-33 |  | -0.39 | <0.05 |
| VSTM2A | 0.3602489 | 4.925E-08 |  | 0.4881056 | 1.6E-17 |  | -0.610727 | 8.066E-33 |  | -5.039728 | 2.231E-90 |  | -0.39 | <0.05 |
| TXNIP | 0.072972 | 0.001181 |  | 0.2518096 | 3.2E-11 |  | -1.000795 | 2.035E-07 |  | -1.440965 | 6.66E-32 |  | -0.387 | <0.05 |
| FBLN1 | 0.221191 | 5.789E-09 |  | 0.5992732 | 1E-15 |  | -0.534768 | 0.0013404 |  | -1.588631 | 3.298E-18 |  | -0.384 | <0.05 |
| GOLM1 | 0.1284043 | 8.334E-06 |  | 0.4679409 | 6.2E-18 |  | -0.813639 | 1.205E-12 |  | -1.158101 | 4.556E-25 |  | -0.384 | <0.05 |
| LRRN4CL | 0.1197135 | 3.356E-10 |  | 0.1800836 | 5.4E-15 |  | -0.508313 | 0.0017606 |  | -1.792089 | 1.283E-35 |  | -0.383 | <0.05 |
| RCSD1 | 0.1222921 | 4.007E-05 |  | 0.124396 | 1.1E-09 |  | -0.725431 | 6.992E-05 |  | -1.595978 | 1.858E-34 |  | -0.382 | <0.05 |
| TRIM9 | 0.2899037 | 1.367E-10 |  | 0.4325468 | 9.5E-17 |  | -0.604876 | 1.112E-23 |  | -2.303759 | 7.288E-31 |  | -0.38 | <0.05 |
| FCGBP | 0.0391644 | 8.334E-06 |  | 0.0361772 | 5.7E-05 |  | -4.296207 | 3.854E-13 |  | -2.231364 | 1.65E-15 |  | -0.378 | <0.05 |
| ARRB1 | 0.0840106 | 3.096E-06 |  | 0.1520375 | 9.4E-17 |  | -0.832778 | 5.637E-19 |  | -1.038429 | 1.085E-29 |  | -0.374 | <0.05 |
| STMN2 | 0.1618245 | 0.0005859 |  | 0.2814341 | 2E-16 |  | -2.306116 | 3.989E-20 |  | -3.437133 | 1.752E-66 |  | -0.374 | <0.05 |
| BIN2 | 0.0424554 | 0.0021086 |  | 0.116557 | 9.4E-11 |  | -0.565985 | 1.98E-07 |  | -1.07498 | 3.621E-22 |  | -0.37 | <0.05 |
| NKX2-3 | 0.8116456 | 8.379E-12 |  | 1.2762639 | 4.8E-19 |  | -1.32648 | 6.719E-08 |  | -2.719296 | 2.773E-80 |  | -0.365 | <0.05 |
| CPNE5 | 0.1152519 | 4.456E-08 |  | 0.1969204 | 2.1E-17 |  | -1.215399 | 9.938E-17 |  | -1.971032 | 8.658E-50 |  | -0.362 | <0.05 |
| MAL | 0.6516369 | 3.899E-13 |  | 0.2342931 | 2.7E-10 |  | -0.530508 | 5.359E-08 |  | -3.37398 | 2.667E-78 |  | -0.359 | <0.05 |
| ARHGAP20 | 0.9675783 | 1.963E-12 |  | 1.4833398 | 8.3E-20 |  | -0.63462 | 6.166E-29 |  | -2.477524 | 1.545E-46 |  | -0.358 | <0.05 |
| BEX1 | 0.2086147 | 6.798E-06 |  | 0.2173287 | 1.5E-11 |  | -0.811647 | 8.213E-08 |  | -2.423093 | 3.077E-18 |  | -0.358 | <0.05 |
| MARCKS | 0.2001844 | 8.334E-06 |  | 0.4263463 | 2.4E-06 |  | -1.051639 | 2.434E-16 |  | -1.088959 | 1.032E-30 |  | -0.357 | <0.05 |
| LEAP2 | 0.0693631 | 0.0069304 |  | 0.0647455 | 0.01777 |  | -1.498474 | 1.175E-18 |  | -1.323788 | 3.91E-25 |  | -0.356 | <0.05 |
| C1orf115 | 0.1359347 | 0.025549 |  | 0.134109 | 0.01183 |  | -1.366436 | 4.664E-32 |  | -2.360664 | 1.042E-83 |  | -0.352 | <0.05 |
| CKB | 0.3330021 | 2.222E-07 |  | 0.5401402 | 1.2E-14 |  | -2.506807 | 4.21E-13 |  | -1.365303 | 3.274E-08 |  | -0.352 | <0.05 |
| ADAMTS1 | 0.5495539 | 9.662E-08 |  | 0.8459353 | 2E-16 |  | -0.5137 | 0.0335973 |  | -1.827032 | 5.255E-38 |  | -0.344 | <0.05 |
| CD177 | 0.0694484 | 0.0102187 |  | 0.151803 | 2.3E-07 |  | -4.909569 | 3.976E-50 |  | -5.629963 | 3.67E-110 |  | -0.341 | <0.05 |
| CBLN2 | 0.4496593 | 7.128E-13 |  | 0.7365467 | 9.2E-20 |  | -0.795627 | 9.6E-25 |  | -2.77379 | 4.655E-49 |  | -0.338 | <0.05 |
| DPT | 0.0351053 | 8.004E-05 |  | 0.0930444 | 1.3E-10 |  | -1.707026 | 2.055E-11 |  | -3.817686 | 1.287E-88 |  | -0.337 | <0.05 |
| C16orf62 | 0.0897938 | 6.562E-05 |  | 0.1651934 | 2.9E-11 |  | -0.614643 | 4.3E-11 |  | -1.226084 | 7.066E-20 |  | -0.334 | <0.05 |
| GPRIN2 | 0.2098406 | 1.679E-09 |  | 0.2228121 | 2.6E-14 |  | -1.217944 | 1.226E-31 |  | -1.367496 | 2.254E-18 |  | -0.333 | <0.05 |
| USP2 | 0.1553284 | 1.001E-06 |  | 0.3614564 | 1.4E-08 |  | -0.721028 | 9.613E-38 |  | -4.024403 | 2.09E-168 |  | -0.332 | <0.05 |
| RELN | 0.2845508 | 9.787E-10 |  | 0.616346 | 1E-17 |  | -0.637742 | 1.348E-09 |  | -2.483781 | 2.863E-28 |  | -0.33 | <0.05 |
| CHRDL1 | 0.5546954 | 2.316E-06 |  | 0.7287674 | 3.7E-11 |  | -2.409466 | 5.399E-13 |  | -2.914359 | 2.28E-30 |  | -0.328 | <0.05 |
| SYNM | 0.0958787 | 0.025549 |  | 0.1066067 | 1.2E-08 |  | -0.991138 | 0.0192345 |  | -4.204915 | 3.989E-87 |  | -0.325 | <0.05 |
| FEV | 0.5652078 | 1.407E-09 |  | 0.9368735 | 7.5E-16 |  | -0.617265 | 2.562E-16 |  | -3.696252 | 5.16E-37 |  | -0.318 | <0.05 |
| LAMA1 | 0.1773978 | 1.127E-09 |  | 0.2779157 | 6.2E-15 |  | -1.972363 | 1.589E-40 |  | -2.389538 | 7.286E-43 |  | -0.315 | <0.05 |
| EYA2 | 0.2253055 | 0.0025126 |  | 0.3747036 | 0.00038 |  | -1.283088 | 5.711E-11 |  | -2.782979 | 1.565E-35 |  | -0.314 | <0.05 |
| NEURL1B | 0.0632448 | 2.047E-06 |  | 0.0857278 | 7.2E-12 |  | -1.25902 | 4.755E-13 |  | -1.482258 | 4.439E-47 |  | -0.31 | <0.05 |
| GSTM2 | 0.5616024 | 4.708E-11 |  | 0.7581245 | 1.9E-19 |  | -0.648707 | 1.633E-05 |  | -1.338918 | 4.226E-23 |  | -0.308 | <0.05 |
| GPX3 | 0.1186476 | 0.039909 |  | 0.2854633 | 1.1E-08 |  | -1.727765 | 2.394E-09 |  | -2.254155 | 2.535E-42 |  | -0.307 | <0.05 |
| SUCLG2 | 0.0177513 | 0.046721 |  | 0.1644111 | 3.7E-11 |  | -1.364409 | 7.237E-20 |  | -1.249631 | 2.609E-46 |  | -0.306 | <0.05 |
| SH3GL3 | 0.4441919 | 1.257E-07 |  | 0.7260378 | 3.6E-15 |  | -0.78773 | 3.083E-36 |  | -1.651922 | 1.561E-05 |  | -0.304 | <0.05 |
| DPP10 | 0.1192177 | 9.614E-10 |  | 0.2595522 | 1.5E-17 |  | -0.809139 | 6.68E-05 |  | -1.633692 | 7.667E-06 |  | -0.303 | <0.05 |
| BMP2 | 0.4721892 | 3.75E-11 |  | 0.7277449 | 5.1E-19 |  | -2.506228 | 4.662E-23 |  | -1.79864 | 3.142E-39 |  | -0.302 | <0.05 |
| SHISA3 | 0.4681138 | 5.86E-06 |  | 0.7020914 | 6.7E-08 |  | -0.638976 | 4.437E-07 |  | -2.065741 | 4.904E-18 |  | -0.302 | <0.05 |
| CXCL12 | 0.1782781 | 0.0344656 |  | 0.306633 | 3.3E-05 |  | -1.385496 | 1.029E-07 |  | -2.488271 | 3.978E-63 |  | -0.301 | <0.05 |
