# Supplementary material for: Development and validation of a risk prediction model and nomogram for colon adenocarcinoma based on methylation-driven genes
Source: Aging (Albany NY). 2021 Jun 28;13(12):16600–19. doi: 10.18632/aging.203179 (PMC8266312; doi:10.18632/aging.203179)
Supplement: Supplementary Tables 2, 3, 4, 5 and 6 [file aging-13-203179-s003.pdf]

SUPPLEMENTARY TABLES

Supplementary Table 2. KEGG pathway analysis for *CBLN2* by GSEA.

Expression	GS follow link to MSigDB	SIZE	NES	NOM p-vale	RANK AT MAX
High	KEGG_RENIN_ANGIOTENSIN_SYSTEM	17	2.04	0	2832
	KEGG_NEUROACTIVE_LIGAND_RECEPTOR_INTERACTION	233	1.72	0.006	3336
	KEGG_TGF_BETA_SIGNALING_PATHWAY	85	1.64	0.012	4441
	KEGG_PENTOSE_AND_GLUCURONATE_INTERCONVERSIONS	26	1.72	0.02	1987
	KEGG_CALCIUM_SIGNALING_PATHWAY	170	1.58	0.029	3276
	KEGG_THYROID_CANCER	29	1.5	0.042	2089
	KEGG_HEDGEHOG_SIGNALING_PATHWAY	54	1.53	0.049	2207
Low	KEGG_HOMOLOGOUS_RECOMBINATION	26	-1.81	0.014	3924
	KEGG_NUCLEOTIDE_EXCISION_REPAIR	44	-1.74	0.014	2907
	KEGG_RNA_POLYMERASE	28	-1.69	0.023	3300
	KEGG_PROTEIN_EXPORT	23	-1.65	0.028	3135
	KEGG_PYRIMIDINE_METABOLISM	96	-1.68	0.034	4569
	KEGG_SPLICEOSOME	126	-1.72	0.036	4531
	KEGG_ONE_CARBON_POOL_BY_FOLATE	17	-1.72	0.039	1513
	KEGG_ANTIGEN_PROCESSING_AND_PRESENTATION	66	-1.69	0.049	4709

Supplementary Table 3. KEGG pathway analysis for *RBM47* by GSEA.

Expression	GS follow link to MSigDB	SIZE	NES	NOM p-vale	RANK AT MAX
High	KEGG_LONG_TERM_POTENTIATION	67	1.89	0	2756
	KEGG_LYSINE_DEGRADATION	42	1.85	0.002	3723
	KEGG_VASOPRESSIN_REGULATED_WATER_REABSORPTION	42	1.63	0.004	1880
	KEGG_VALINE_LEUCINE_AND_ISOLEUCINE_DEGRADATION	43	1.89	0.006	2684
	KEGG_PROPANOATE_METABOLISM	31	1.87	0.006	4217
	KEGG_FATTY_ACID_METABOLISM	41	1.84	0.006	4437
	KEGG_STARCH_AND_SUCROSE_METABOLISM	46	1.73	0.006	5073
	KEGG_OOCYTE_MEIOSIS	107	1.77	0.008	3220
	KEGG_TERPENOID_BACKBONE_BIOSYNTHESIS	14	1.75	0.008	3277
	KEGG_COLORECTAL_CANCER	62	1.63	0.008	2099
	KEGG_ONE_CARBON_POOL_BY_FOLATE	17	1.77	0.01	3855
	KEGG_BUTANOATE_METABOLISM	32	1.79	0.012	2779
	KEGG_CITRATE_CYCLE_TCA_CYCLE	29	1.75	0.012	3918
	KEGG_THYROID_CANCER	29	1.72	0.012	1389
	KEGG_CYSTEINE_AND_METHIONINE_METABOLISM	33	1.64	0.014	3674
	KEGG_ASCORBATE_AND_ALDARATE_METABOLISM	23	1.82	0.016	5073
	KEGG_PEROXISOME	77	1.74	0.019	3100
	KEGG_AMINO_SUGAR_AND_NUCLEOTIDE_SUGAR_METABOLISM	42	1.67	0.022	2351
	KEGG_P53_SIGNALING_PATHWAY	67	1.56	0.023	3298
	KEGG_APOPTOSIS	84	1.61	0.024	3925
	KEGG_UBIQUITIN_MEDIATED_PROTEOLYSIS	128	1.65	0.027	3522
	KEGG_INSULIN_SIGNALING_PATHWAY	132	1.5	0.031	4333
	KEGG_CHRONIC_MYELOID_LEUKEMIA	73	1.5	0.031	1910
	KEGG_PYRUVATE_METABOLISM	38	1.59	0.034	4217
	KEGG_BETA_ALANINE_METABOLISM	22	1.56	0.037	2684
	KEGG_GLYOXYLATE_AND_DICARBOXYLATE_METABOLISM	15	1.55	0.037	3855
Low	KEGG COMPLEMENT AND COAGULATION CASCADES	64	-1.96	0	3693
	KEGG GLYCOSAMINOGLYCAN BIOSYNTHESIS CHONDROITIN SULFATE	22	-1.79	0.006	3857
	KEGG ECM RECEPTOR INTERACTION	83	-1.83	0.011	3182

Supplementary Table 4. KEGG pathway analysis for *SLCO4C1* by GSEA.

Expression	GS follow link to MSigDB	SIZE	NES	NOM p-vale	RANK AT MAX
High	KEGG_NEUROACTIVE_LIGAND_RECEPTOR_INTERACTION	233	1.81	0.004	4747
	KEGG_JAK_STAT_SIGNALING_PATHWAY	131	1.71	0.012	2903
	KEGG_CYTOKINE_CYTOKINE_RECEPTOR_INTERACTION	235	1.8	0.014	5129
	KEGG_COMPLEMENT_AND_COAGULATION_CASCADES	64	1.74	0.018	4341
	KEGG_HEMATOPOIETIC_CELL_LINEAGE	80	1.77	0.023	3856
	KEGG_CALCIUM_SIGNALING_PATHWAY	170	1.59	0.025	1598
	KEGG_ALDOSTERONE_REGULATED_SODIUM_REABSORPTION	40	1.55	0.026	2604
	KEGG_LEUKOCYTE_TRANSENDOTHELIAL_MIGRATION	110	1.63	0.028	3687
	KEGG_ABC_TRANSPORTERS	43	1.54	0.04	1870
	KEGG_CHEMOKINE_SIGNALING_PATHWAY	182	1.59	0.047	4352
Low	KEGG_SPLICEOSOME	126	-2	0.002	4611
	KEGG_RNA_POLYMERASE	28	-1.89	0.004	4331
	KEGG_BASE_EXCISION_REPAIR	33	-1.79	0.004	3245
	KEGG_PYRIMIDINE_METABOLISM	96	-1.73	0.016	4447
	KEGG_NUCLEOTIDE_EXCISION_REPAIR	44	-1.71	0.017	3718
	KEGG_RIBOSOME	87	-1.76	0.023	3104
	KEGG_PENTOSE_PHOSPHATE_PATHWAY	26	-1.58	0.043	3562

Supplementary Table 5. KEGG pathway analysis for *TMEM220* by GSEA.

Expression	GS follow link to MSigDB	SIZE	NES	NOM p-vale	RANK AT MAX
High	KEGG_CALCIUM_SIGNALING_PATHWAY	170	1.97	0	4043
	KEGG_NEUROACTIVE_LIGAND_RECEPTOR_INTERACTION	233	1.88	0	4071
	KEGG_LONG_TERM_POTENTIATION	67	1.8	0.002	3455
	KEGG_DRUG_METABOLISM_CYTOCHROME_P450	63	1.76	0.007	5236
	KEGG_VASCULAR_SMOOTH_MUSCLE_CONTRACTION	110	1.75	0.007	2795
	KEGG_ETHER_LIPID_METABOLISM	32	1.65	0.01	1224
	KEGG_STEROID_HORMONE_BIOSYNTHESIS	51	1.79	0.011	4850
	KEGG_HEMATOPOIETIC_CELL_LINEAGE	80	1.75	0.016	3753
	KEGG_LEUKOCYTE_TRANSENDOTHELIAL_MIGRATION	110	1.67	0.016	3995
	KEGG_COMPLEMENT_AND_COAGULATION_CASCADES	64	1.74	0.019	4212
	KEGG_NITROGEN_METABOLISM	23	1.67	0.019	3231
	KEGG_GLYCOSPHINGOLIPID_BIOSYNTHESIS_GANGLIO_SERIES	15	1.63	0.025	2056
	KEGG_LONG_TERM_DEPRESSION	64	1.55	0.029	3150
	KEGG_CYTOKINE_CYTOKINE_RECEPTOR_INTERACTION	235	1.67	0.034	6138
	KEGG_HYPERTROPHIC_CARDIOMYOPATHY_HCM	79	1.56	0.035	4984
	KEGG_ALDOSTERONE_REGULATED_SODIUM_REABSORPTION	40	1.53	0.035	3215
	KEGG_TASTE_TRANSDUCTION	40	1.65	0.037	3718
Low	KEGG_NON_HOMOLOGOUS_END_JOINING	11	-1.82	0.002	3947
	KEGG_AMINOACYL_TRNA_BIOSYNTHESIS	22	-1.85	0.004	2245
	KEGG_SPLICEOSOME	126	-2.05	0.006	3198
	KEGG_RNA_POLYMERASE	28	-1.8	0.01	3617
	KEGG_NUCLEOTIDE_EXCISION_REPAIR	44	-1.81	0.014	4043
	KEGG_CELL_CYCLE	124	-1.84	0.016	4208
	KEGG_BASAL_TRANSCRIPTION_FACTORS	34	-1.72	0.022	3845
	KEGG_BASE_EXCISION_REPAIR	33	-1.68	0.022	4083
	KEGG_RNA_DEGRADATION	53	-1.63	0.029	5314
	KEGG_GLYOXYLATE_AND_DICARBOXYLATE_METABOLISM	15	-1.63	0.029	2112

Supplementary Table 6. Primers used in this study.

variable	Gene name	Primer	5'-3'Sequence	size
qPCR	TMEM220	Forward	AGATGCAGAGGTGTGGGTG	169bp
		Reverse	ACGATGCAAGAGGTAGGACG	
	CBLN2	Forward	GCACCATGACCATCTATTTCGAC	266bp
		Reverse	ATGCACTTTGTCTTCCCTTTCC	
	SLCO4C1	Forward	GGAGTTGCACTTACGCTGAG	237bp
		Reverse	CTTTGGCTTCCTGTGTGCAA	
MSP	TMEM220 M	Forward	TAAGGTATCGAAATCGAGGC	141bp
		Reverse	CAACGCTAACGCCATAACT	
	TMEM220 U	Forward	TTTTAAGGTATTGAAATTGAGGT	141bp
		Reverse	CCACAACACTAACACCATAACT	
	CBLN2 M	Forward	TGTGTAAACGTTGTGTCGAC	107bp
		Reverse	CGCCTAATTTTCCGAATCT	
	CBLN2 U	Forward	GTTTGTGTAAATGTTGTGTTGAT	107bp
		Reverse	CCACCTAATTTTCCAAATCTTC	
